# Effects of hypohydration and fluid balance in athletes' cognitive performance: a systematic review

**DOI:** 10.4314/ahs.v22i1.45

**Published:** 2022-03

**Authors:** Adiele Dube, Chantell Gouws, Gerrit Breukelman

**Affiliations:** University of Zululand, Human Movement Sciences

**Keywords:** Hypohydration, cognition, mood, fluid replenishment

## Abstract

**Background:**

The effects of progressive body fluid loss on athletic and cognitive performance are known to result from exposure to environmental heat stress, morphologic factors, and limited fluid replenishment. Athletes need to restore lost body water. However, athletes may fail to maintain euhydration during exercise. This systematic review investigated hypohydration and fluid balance effects on an athlete's cognitive function.

**Methods:**

The PubMed, Sports Discuss, and Ebsco databases were searched for studies reporting on hypohydration, fluid balance and heat on cognitive performance in sport. Multiple phrases including hydration, dehydration, fluid balance, mood, cognition, vigilance, decision making, and brain were explored. Participants in the studies did either receive fluid or did not receive fluid during exercise.

**Results:**

Twenty-four trials (n=493 participants) from 24 articles met the inclusion criteria. Significant hypohydration, >2% body mass loss was reported consistently in 16 publications. Five articles where hypohydration was associated with heat stress and limited fluid intake (3–5% body mass loss) impaired cognitive performance. Mood disturbance, fatigue, and ratings of perceived exertion constantly complemented hypohydration impairment on cognition.

**Conclusion:**

Findings show that hypohydration impairs cognitive performance and mood at higher levels of 3–5% body mass loss. However, sport-specific cognitive protocols of accessing hypohydration and fluid balance in individual and team sports remain equivocal.

## Introduction

Mega sporting events will continue to take place in diverse hot geographical environment across the globe as they have been in the past and in the present. As always, hypohydration can be expected in these events. The events include the Olympic Games: Beijing 2008; Rio 2016, Tokyo 2021; World Athletics Championships Doha 2019, and Federation International Football Association World Championships Qatar 2022 1,2. With such exposure to hot environments, athletes mainly in prolonged vigorous exercise, racket and intermittent team sports experience significant and exceeding >2% body fluid loss due to thermoregulation 2,3. Inadequate and/or no fluid loss replacement can cause endurance capacity impairment associatd with physiological and cognitive function alterations^4^,^5^. Indeed excessive dehydration impacts are major cause of concern to athletic trainers and sports medical staff.

Dehydration and hypohydration deleterious effects on athletic performance and cognition have been widely researched^1^,^3^,^5^. It is well known that a 2% body mass loss can impair endurance performance in humid/hot environments^6^,^7^. There has been limited research on the impact of hypohydration on athlete's cognitive performance and mood during individual and intermittent team sport^8^,^9^. Literature has supported that dehydration may impair cognitive performance^10^- ^12^ and functional task^13^. However, it is known that rehydration may cause minimal or no effect on athletic, cognition and immunological performance if the outcome to be assessed is insensitive to the modest (up to 2% of body weight) fluid losses^1^, ^5^, ^8^. Severe dehydration may disturb, aggravated fatigue, dizziness, confusion and often severe cases lead to delirium, coma and death^14^–^19^. Various studies have demonstrated that heat-stress and exercise-induced dehydration did not alter cognitive performance^4^, ^11^, ^20^, ^21^. However, inconsistent conclusions exist in the current literature^4^, ^5^, ^8^, ^12^. Some studies have demonstrated discrepancies in literature may be due to task complexity, test duration, magnitude of heat stress, test combined^11^, ^13^.

Prolonged exercising in hot, humid environments with inadequate fluid replenishment may increase core body temperature (hyperthermia) to ∼4°C provoking athlete's mental status that worsen in moderately and untrained athletes^4^, ^22^, ^23^. Despite that elite acclimated athletes may physiologically negotiate hyperthermic conditions, athletic trainers, sports scientists and sports medical staffs tirelessly work to uncover cooling techniques to curb core body temperature, delayed onset peripheral and central fatigue^4^, ^23^–^26^. Thus, researchers have investigated dehydration, hypohydration and fluid ingestion aspects and their subsequent athletic performance effects^2^,^8^ remains unclear. To date, no papers have reviewed and collectively discussed these aspects to equip professionals better understand impact on individual and team sport performances. Therefore, the aim of this systematic review was to summarise to summarise the literature assessing impact on hypohydration and fluid balance in relation to cognitive function in semi-professional to elite athletes exercising in humid, hot environments.

## Methods

The study protocol was devised following the specifications outlined in the Preferred Reporting Items for Systematic Reviews and Meta-analysis (PRISMA) Statement^27^.

### Literature Search strategy

Relevant research studies on dehydration and hypohydration effects on cognitive function when training in hot, humid environments identified on electronic database from 2005 until May 2020. Available literature before 2005 focused mainly on athletic performance of elite athletes. For the purpose of the current review focus was on cognitive function and mood of semi-professional compared to elite athletes. The database include: PubMed/Medline, Sports Discuss, and Ebsco. Keywords and terms search were hydration, athletic, exercise, mood, attention, vigilance, decision making, reaction time, sweat loss, individual/team sport, ad libitum, water, fluid (eg. administration, consumption, ingestion, intake, replacement, replenishment), hydration (de-, eu-, hypo-, re-), each combined with cognitive/cognition (aspects, function), brain were explored. Searches were restricted to full-length peer-reviewed published articles in the English language. Unpublished experimental observations, published abstracts records that contain irrelevant terms (children, elderly, patient, disease, rat/mouse) were excluded. Twenty four original research studies involving fluid balance (water, flavoured water/ ad libitum water/ sodium chloride solution/ sports drink) not controlled by investigators, and sweat rate in athletes were included. These studies measured impact of hypohydration on cognitive performance. Little attention was paid to effects of physical/athletic performance considering that the data was available and up to date.

### Inclusion

All research studies with fulfilling the following criteria were eligible for inclusion;

- All relevant South Africa and international studies.
- Studies with standardized dehydration protocol.
- Focussed on male or female humans with no underlying medical conditions (≥ 13 years of age).
- Fluid consumption was done in limited time ≤ 4 hours between dehydration protocol and subsequent performance test.
- A cognitive function and athletic performance outcome measured.

### Exclusion

- Unpublished experimental observations, published abstracts records that contain irrelevant terms (elderly, patient, disease, and rat/mouse) were excluded.
- Experimental designs without cognitive performance outcome measured.

### Data Extraction

All published scientific research peer-reviewed articles meeting the inclusion criteria were extracted and considered for the following characteristics; participant, exercise and hydration protocol, change in body mass, study findings, and study limitations. Research studies that contained more than one intervention and eligible for inclusion tested cognitive performance under two different conditions were treated as separate trials^16^. These trials derived from one study are denoted and cited by letters (a-b). Where necessary information was not given, the author considered it as unavailable.

### Fluid balance terminology

An athlete is responsible for maintaining normal hydration status (euhydration) for optimal body performance. Armstrong^28^ suggests that change in body fluid balance is a resultant of baseline mass value compared to the individual body mass. Hydration status can be explained using terms such as; euhydration (normal baseline body water content), hypohydration (excessive body water deficit) and dehydration (progressive body water loss from normal baseline to hypohydration).

### Search results

**Fig. 1:**
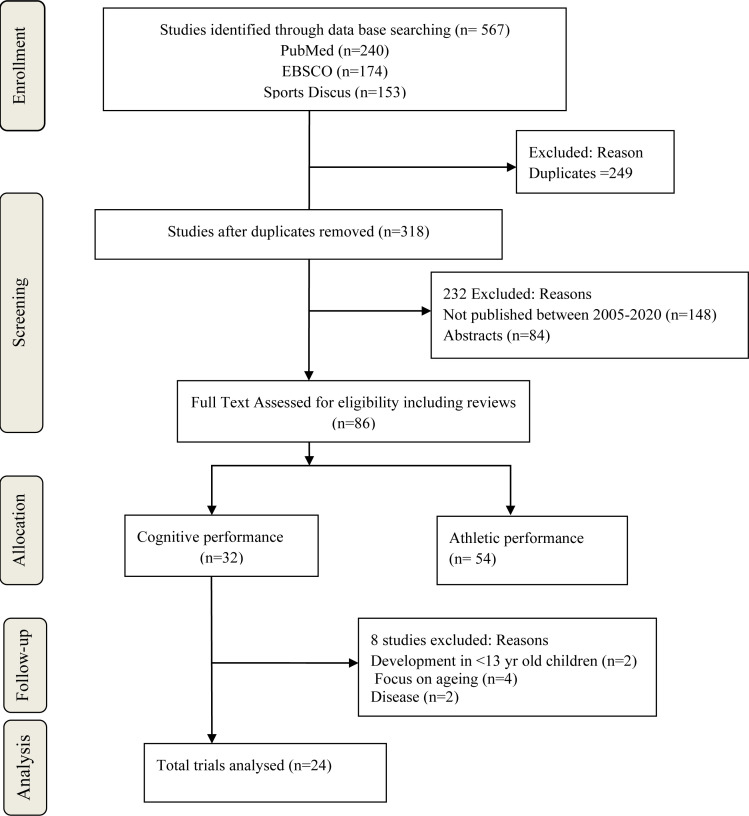
PRISMA Flow Chart of study selection process

567 articles were potentially relevant. After the exclusion of duplicates, articles published before 2005, articles focusing on athletic performance, ageing, diseases and children below 13 years and review of full-text versions, 24 articles were selected for review as shown in Fig 1 and Table 1 below.

**Table 1:** Summary of research studies evaluating effects hydration levels on cognitive performance

Citation	Participants	Protocol	Hydration loss levels (% Δ body mass	Fluid Type	Key Findings			Limitations
					Physiological and subjective measures	Cognitive domains assessed	Hyperthermia and Hypohydration effects on cognition	
Goodman et al. (29)	N=15 12M, 3 Gender not revealed Military Defence Force 21–34yrs USA	Crossover designed, 90min self- paced military march in standardised military attire in the heat with a 20kg backpack Fluid restriction and or prescribed fluid intake throughout the exercise. Cognitive test battery Envir. Conditions: 39.5–41.8°C, 28–42% RH	2.28 (HYP trial- no fluid) 0.53 (EUY trial – Ad libitum fluid)	Ad libitum water	HYP: ↑Core body temp. ↑HR, ↑RPE, ↑Thirst. EUY: No significant difference in core body temp, perceived thirst	Information processing, memory, impulsivity, attention, and concentration, response time domains	HYP: ↓working memory ↓response times ↓attention task ↓depression ↓accuracy No significant effect on immediate or delayed memory, accuracy, and response speed.	Participants not blinded to hydration status
Wittbrodt et al. (30)	N=13 7M, 6F 19–28yrs Healthy recreationally active adults USA	2-week; Counterbalanced 150min trial (intermittent exercise protocol): Three experimental; no exercise heat stress (CON), exercise heat seat stress with fluid replacement (EUY), exercise heat stress with dehydration (DEHY), exercise heat stress without fluid replacement (HYP) Visuomotor Pacing Task (VMPT) Envir. Conditions: EUY, HYD, HYP : 45°C, 15% RH CON: 22 °C, 30% RH	3.1 DEHY, HYP (3.3 men 3.1 women) 0.2 EUY 0.0. CON	EUY, DEHY: water equivalent to sweat HYP: No fluid, only mouth rinse once per hour	↑HR, ↑Rectal temp., RPE, Thirst	Visuomotor functioning, Accuracy, reaction time	EUY, DEHY: Visuomotor performance impaired A significant effect on processing accuracy, and reaction time	Participants not blinded to hydration status
MacLeod et al. (1)	N=8F 19–22yrs Healthy unacclimatized elite hockey players UK	4 experimental sessions: 50min Hockey intermittent treadmill protocol with prescribed fluid intake to replace sweat loss; ad libitum water intake, or no fluid Cognitive testing after treadmill protocol Envir. conditions: Hot; 33.2–33.4°C, 58–60% RH Moderate; 13–19°C, 51–55% RH	HYP: ∼2 no fluid EUY: ∼ 0.0 No difference in ad water intake on moderate temp.	Ad libitum water	↑RPE ↑Thirst (HYP) prior to treadmill protocol No significant effect on HR and Temp (body core)	Process speed, working memory, perceptive discrimination, visual scanning/ processing speed	HYP: ↑Psychomotor function, visual scanning/ process speed EUY; ↑ working memory	Participants not blinded to hydration status
Piil et al. (13)	N=139 139M 30–32yrs Recreationally active Cyprus, Denmark, Greece, Spain (Compiled in Greece)	Laboratory experiment: (EUY, DEHY) Occupational study (urine sampling), 8M for laboratory experiment in an environmental chamber with fluid replacement Motor-cognitive test battery pre- & post- Envir conditions: Manufacturing; 29°C, 25% H Agriculture; 29 °C, 55% RH Police officers; 27 °C, 50% RH Tourism; 30 °C, 55% RH Construction; 26 °C, 54% RH Environmental chamber; 40 °C, 25% RH	∼ 2.0 (no fluid) 0.0 (fluid replacement)	Water	↑RPE, ↑Core body temp., ↑Thermal comfort, ↑Thirst ↑HR	Process speed, working visual scanning/ processing speed	No significant effect on cognitive domains	Participants unaware of the researcher's hypothesis and naive to the purpose of the studies
Van den Heuvel et al. (31)	N=17 17M 25yrs Healthy, non-smoking Australia	Three Passive thermal-hydration protocol (water immersion) with states and then clamped using controlled, isotonic fluid administration. Unique immersion protocol establishment in the first trial and replicated in subsequent trials averaging 185min (137–242min) Envir. Conditions: Temperate; 34–35 °C, Warm water; 40–41 °C	3 and 5 (HYP) 0.0 (EUY trial)	sodium chloride NaCI+	↑HR, Thermal state, Core body temp., in HYP at 3% and 5%	Visual perception, working memory	↑Decision process modified ↓Depression ↓Discriminative ability (hyperthermia) No significant effect visual and working memory following 3–5% dehydration	Participants not blinded to hydration status
Gamage et al. (32)	N=30 30M 22yrs, elite cricketers UK	Fluid restriction(4ml/kg/h) or fluid provision (12–15 ml/kg/h) during 2h of standardised cricket training Envir Conditions: Outdoors: 27.2–32.8 -°C89, R66H, ∼2mph wind speed	3.7 fluid restriction trial 0.9 fluid provision trial	Not reported	Not reported	Process speed, working memory, perceptive discrimination, visual scanning/ processing speed	Not reported	Participants not blinded to hydration status Fluid type unknown No validity or reliability testing of sport (cricket) skill
Wittbrodt et al. (33)	N=12 12M Recreational active USA	Vigorous exercise intensity for 50mins Fluid assimilation time >50min Envir Conditions: Ambient temp 32°C, 65 RH	1.5	Water	↑ HR ↑altered skin temp. ↑ Thirst, ↑ fatigue	Process speed, working memory, perceptive discrimination, visual scanning/ processing speed	No effect	Participants not blinded to hydration status Exercise intensity not mentioned
Wilson et al. (34)	N=8 8M Licensed jockeys UK	Exercise for 45 minutes Fluid assimilation time ∼35min	1.8	Water	Not reported	Response inhibition	No effect	Participants not blinded to hydration status
Owen et al. (35)	N=13 13M 22 yr olds, soccer semi- professional players UK	LIST protocol (90mims) with prescribed fluid intake to replace 89 sweat loss; ad libitum, water intake, or no fluid LSST and LLSPT performed after LIST protocol Envir Conditions: 19.4°C, 59.4 RH	0.3 (water intake) 1.1 (ad libitum water) 2.5 (no fluid)	Ad libitum water	↑ RPE (no fluid than water intake) ↑HR (no fluid than water intake and ad libitum water)	Process speed, working memory, perceptive discrimination, visual scanning/processing speed	No effect	Participants were not blinded to hydration status
MacLeod et al. (3)	N=8 8F 22yr olds, Elite field hockey players UK	2-day experiment Day 1: Baseline hockey skill measurement Passive heat stress (39.9 °C, 73 RH) → controlled fluid intake to induce HYPO or EUH Day 2: 60 min-hockey imitated and designed intermittent treadmill protocol Hockey skills test in a gymnasium Envir Conditions: Treadmill protocol; 33.3 °C, 59 RH Gym 16.3 °C 22.2 °C,	∼ 2 (HYP trial) Day 2: ∼ 0 (EUY trial) No difference in fluid intake Replacement fluid loss (88 vs 80) %	Ad libitum water	↑RPE and ↑Thirst (HYPO) prior to treadmill protocol No significant effect on HR and Temp (body core)	Process speed, working memory, perceptive discrimination, visual scanning/ processing speed	↓ decision making time (skills test) ∼7 slower (HYP vs EUY) prior to treadmill protocol No significant effect on decision making time post treadmill protocol	Protocol, not field sportspecific but intermittent treadmill protocol Use of Day 1 passive heat stress for Day 2 trials may be invalid Participants not blinded to hydration status
Hoffman et al. (36)	N=10 10F 21 yr division 1 college Basketball player	USA 40 min live scrimmage exercise Quick board lower body reaction agility, Dynavision D2- visual reaction time – all performed prior and post live scrimmage Envir Conditions: Indoors 22.6°C, 50.9 RH	2.3 no fluid) Not availed (water intake)	Water	No significant effect on HR and player load	Psychomotor function/process speed, visual scanning/processing speed	No significant effect on visual reaction time	Participants not blinded to hydration status No trial report for Δ body mass during water intake Cognitive tests not validated prior
Brandenburg & Gaetz (37)	N=12 12F 24yr Basketball Elite players Canada	A descriptive study covering 2 international indoor matches Envir Conditions: 22.5 – 23.5 °C 44–50 RH	1^st^ match -2.1 to +5 2^nd^ match -2 to +0.1	Diluted ad libitum and water according to individual taste	↑ HR	Process speed, working memory, perceptive discrimination, visual scanning/processing speed	No significant effect on field goal percentage Adverse relation (goal vs body mass loss in the 2^nd^ match	Carbohydrate has the confounding potential effect on Goal percentage No controlled trial (EUY)
Ely et al. (38)	N=32 32M Healthy and non-heat	3-week experiment EUY and HYP trials, 3h work-rest cycle,	4	Sodium chloride (NaCI) + water	HYP (no fluid replacement)	Psychomotor function/process	No significant effect on mood and cognition	Carbohydrate ingestion may have confounding
Carvalho et al. (39)	N=12 12M 14–15yr Basketball national team players Portugal	90 min training session HYP trials Basketball drills before and after training Envir Conditions: Indoors; 21.9–26.0 -°C 5,4 4.18 .3 RH	2.5 (no fluid) 1.1 fluid intake)	Ad libitum water	HYP trial: ↑RPE in	Process speed, working memory, perceptive discrimination, visual scanning/processing speed	Not availed	Participants not blinded to hydration status EUY (control) trial not available Basketball drill not validated prior
Ali et al. (40)	N=10 10F Soccer Premier division players New Zealand	90min LIST protocol with fluid intake (15ml/kg) or without LSPT performed before, during, and after LIST Envir Conditions Not availed	2.2 (HYP) 1.0 (EUY)	Water	HYP trial: ↑RPE, core temperature, HR, blood lactate	Processing speed, perceptive discrimination, visual scanning	No significant effect; perceived activation and (dis- pleasure)	Participants not blinded to hydration status EUY (control) trial not available
Giano et al. (17)	N=24 24M Physically fit USA	3-day laboratory experiment. DEHY + Diuretic DEHY + Placebo EUY + Placebo Envir Conditions: 26.1–27.9 °C, 54 Wind speed	1.59	Water	HYP trial: ↑RPE, core temp, HR	Process speed, working memory, perceptive discrimination, visual scanning/ processing speed	↑Processing speed and working memory ↓Fatigue	Participants were not blinded to hydration status
Bandelow et al. (21)	N=20 20M University soccer players UK	Cognitive battery tests: Sternberg test Corsi block test, Finger tapping test Visual sensitivity test Trials before, at half-time, after the match HYP Envir Conditions: 34°C, 62 -65 RH	2.5	Ad libitum water Sports drink	Not reported	Process speed, working memory, perceptive discrimination, visual scanning/ processing speed	↓working memory (HYP) No significant in fine motor speed, working memory, reaction time	EUY trial not available (control) No sport-specific cognitive tests
D'Anci et al. (16) a	N=31 16M; 15F University lacrosse and rowing athletes USA	Study 1: HYP trial, EUY trial Coach-run, hard natural practice Cognitive test battery post-practice Envir Conditions; RH not stated Assimilating time 60–70min	2.0 (HYP) 0.1 (EUY)	Water	HYP trial: ↑Thirst, ↑POMS: tension, anger, fatigue, depression ↓ vigor	Vigilance attention, shortterm memory, simple and choice reaction, map planning, visual perception, mathematical addition, mood	HYP: ↑Processing speed ↓Vigilance, depression (3–4%) No effect on spatial memory, reaction time, map planning, mathematical addition	Participants were not blinded to hydration status
D'Anci et al. (16) b	N=24 12M; 12F University lacrosse, rowing, and American football athletes USA	Study 2: HYP trial, EUY trial Coach-run, hard natural practice Cognitive test battery postpractice Envir Conditions RH not stated Assimilating time 60–75min	1.7 (HYP) +0.1 (EUY)	Water	HYP trial: ↑Thirst, ↑POMS: tension, anger, fatigue, depression ↓ vigor	Vigilance attention, short-term memory, simple and choice reaction, map planning, visual perception, mathematical addition, mood	No effect on short-term and spatial memory, reaction time, map planning, mathematical addition	No sport-specific cognitive tests administered
Adam et al. (20)	N=8 8M Active soldiers (6) USA	Heat exposure for 300 min Envir Conditions: 20 °C, 50% RH Wind speed 1 to 2.2 m/s	3.0	No fluid	↑Thirst, thermal discomfort ↑altered skin temp. ↑fatigue ↑HR	Processing speed, working memory, perceptive discrimination, vigilance, visual scanning	No significant effect on cognitive domains	EUY trial not available (control)
Baker et al. (41)	N=11 11M 17–28yr male competitive basketball players USA	Experimental: 3hr interval walking in heat chamber; HYP trials, EUY trials, 80 min stimulated match Attention variables test: baseline, post chamber, post-match Envir Conditions: 40 °C, 20% RH (heat chamber), room temp. (indoor match)	HYP: 1%, 2%, 3%, 4% EUY: 0	No fluid Flavoured water	HYP trial (1–4%): ↑lightheaded, overheat, fatigue No effect of core body temp.	Attention variables, perceptive discrimination, vigilance, visual scanning	HYP trial (1–4%): ↑ commission and omission errors ↓response time (6–8%)	Participants were not blinded to hydration status Rationale of induced heat stress to attention variables test before a basketball match unrealistic
Edwards et al. (42)	N=11 11M moderately active soccer players New Zealand	90 min exercise: 45 min cycling, 45 min soccer match (80 fluid loss replacement) Post-match mental concentration test (number identification) Envir Conditions: 24- 25 -°C 5,5 4 R7H (cycling), 19- 21 -°C57, 4R6H (soccer match)	0.7 (fluid intake) 2.1 (mouth rinse) 2.4 (no fluid)	Water mouth rinse No fluid	↑ HR ↑thermal discomfort ↑altered skin temp. ↑ Thirst, ↑ fatigue	Processing speed, visual scanning	No significant effect on mental concentration	Participants were not blinded to hydration status The rationale of cycling before a match in soccer is doubtful
Serwah & Marino (43)	N=8 8M 25yrs Healthy volunteers Australia	90min discontinuous fixedintensity exercise: 3 experimental conditions (full fluid replacement, half fluid replacement, no fluid Own bicycle mounted on the electromagnetically braked cycle trainer Envir. Conditions: 31.3°C, 62.1–64.5% RH Wind speed 2m/s	2.0 (full fluid) 1.0 (half fluid) 1.7 (no fluid)	Water	No fluid: ↑HR ↑Skin temp., ↑Thirst No effect of core body temp. in full and half fluid conditions	Processing speed, working memory, perceptive discrimination, vigilance, visual scanning	No significant effect on cognitive domains	Participants were not blinded to hydration status No sport can employ a discontinuous fixed-intensity nature of exercise protocol
Szinnai et al (44)	N=17 8M 7F 25–33yrs Health non-smoking volunteers Switzerland	Experimental done in random order EXP; CON Female: Pre and post menstrual Men: Cognitive function test Envir. Conditions: Cognitive tests: 22°C	1.75 CON 3.26 DEHY	Mineral water	No fluid: ↑HR ↑Fatigue ↑Thirst ↓Alertness No significant effect in the control group	Processing speed, working memory, perceptive discrimination, vigilance, visual scanning, reaction time	No significant effect on cognitive domains in moderate dehydration	Participants were not blinded to hydration status

EUY- euhydration, HYD-Hydrated, DEHY- dehydration, HYP-hypohydration, CON- control, Envir. Conditions- environmental conditions, RH. relative humidity, HR- heart rate, N- Number, M- male, F-female, LIST- Loughborough intermittent shuttle test, LSPT-Loughborough soccer passing test, RPE-rate of perceived exertion, Temp- temperature, ↑- increase, ↓- decrease

## Discussion

This systematic review aimed to summarise literature assessing the impact of hypohydration and fluid balance on cognitive function in semi-professional to elite athletes exercising in humid, hot environments. The discussion considered the risk factors posed by an increase in sweat loss to ≥2% body mass loss. Major causes of hypohydration were discussed as environmental factors, exercise intensity, and/ or limited fluid replacement in relation to the brain and cognitive performance. Effects on cognitive performance and mood in the studies included in this review considered individual and team sports with training or competition duration of more than 1 hour 8, 23, 26. Although hypohydration risk levels may vary in different sports, the review takes the notion that individual risk factors among athletes may be altered between low- and high-level categories depending on humidity, timing day/season and intensity level, hydrating behaviours, social and cultural considerations.

### Fluid balance and the Brain

The brain, a complex active part of the human body is known for its high metabolism. It accounts for ∼15% of resting cardiac output and a relatively higher total body aerobic metabolism of ∼20%^45^, ^46^. To maintain its high metabolism, the brain depends solely on adequate circulation of oxygen, metabolic substrates, and metabolic by-products elimination^5^. Heat stress, hyperthermia, and dehydration are known physiological stressors to alter cerebral circulation and metabolism. Hypohydration was found to mediate brain function reduction by reducing cerebral blood flow and brain cell volume, hence increasing blood-brain permeability^8^.

Exercise stimulus causes adjustments to Cerebral Blood Flow (CBF). A study by Kety and Schmidt47 showed that CBF could not be altered during the athletic rest-to-exercise transition. Recent temporal resolution methods showed a ∼20% CBF rise due to endurance and moderate exercise intensities^46^–^49^. Indeed, CBF is subdued with high exercise intensities and significantly surpass rest levels due to exhaustion^50^, ^51^.

Progressive dehydration during individual and/ or intermittent team sports without concomitant hyperthermia increases CBF^52^. However, when the athlete is resting, a 1.5°C increase in body core temperature causes a ∼15% CBF reduction^53^. It should be noted that both dehydration and hyperthermia changes CBF mechanisms in different exercises^5^, intensities, and environments. Dehydration ≥3% body mass loss during endurance exercise in a hot, humid environment reduces CBF due to cerebrovascular instability and cardiovascular drift^54^ – ^56^. In contrast, CBF reduction is attenuated when there is equilibrium between body fluid lost through sweating and fluid replenishment during exercise^5^, ^54^, ^57^.

Heat-induced stress, hyperthermia, and dehydration effects on CBF are associated with prolonged aerobic exercise^5^. Previous studies reveal that CBF reduction is worsened during acute-intensity exercise in hot and humid environments^23^, ^30^, ^51^, ^58^, compared to cold and temperate environments. Similarly, elite athletes' dehydration levels during training or competition in hot environments are compensable despite reduced CBF and work rate than their untrained counterparts. When athletes maintain euhydration status, the mechanisms and dynamics of CBF tend to normalise^51^. Therefore, endurance exercising in a hot and humid environment provokes dehydration, and hyperthermia enhancing cerebrovascular strain with CBF decline.

### Hypohydration and Cognition

Excessive dehydration (hypohydration) effects on cognitive performances have been widely researched across different ages and populations of varied physical fitness. Scientific evidence shows inconsistent findings. Some studies revealed that hypohydration does not affect cognition^20^, ^42^, ^59^ others showed a reduction in cognitive function among military, athletes, young healthy adults, and the elderly^16^, ^17^, ^29^ - ^31^, ^41^, ^60^. Despite evidence of fluid intake benefits on cognitive function observed, literature lacks a clear indication of better treatment efficacy on specific cognitive domains^4^, ^60^. Cheuvront & Kenefick^7^ indicated a lack of clear mechanism by which hypovolemia or hyperosmolality cause cognitive impairment. Studies, however, consistently report hypohydration effects on brain function through 1–4% body mass loss reported in cognitive performance literature^4^, ^23^, ^30^. Prolonged exercise in hot environments without fluid replacement elevates core body temperature thereby creating a cognitive burden^21^. The symptoms of hypohydration including thirst and negative mood states have an equal effect on accomplishing cognitive tasks and consequently impair function^7^, ^8^. Cognitive trials conducted in less than 5 minutes after dehydration protocol ended found that ≤ 2.8% body mass loss induced through fluid deprivation had no impact on cognitive-motor performance^13^, ^40^, ^42^–^44^. Although many studies did not clearly show the time from the end of dehydration protocol to commencement of the cognitive tests, a significant raise in ratings of thirst, concentration, and ratings of perceived effort was found^13^, ^16^, ^42^. In all the above trials, the long-lasting effects of physiological stressors employed may obstruct fluid intake influencing cognitive performance^8^, ^60^.

Fluid replenishment attenuates Total Mood Disturbance in 3 of the 5 trials where mood was measured^16^, ^23^, ^29^, ^31^. Considering that mood effects and cognition were independent, it should be noted that the above three findings were objective compared to the subjective60. However, if not clearly stated, self-reported mood questionnaires are subjective and consider mood effects as dependent variables. It is certain that the influence of fluid replenishment on cognitive function and mood needs further research^23^, ^60^.

It should be noted that rehydration may have no or little effect on cognitive function in cases where outcome measured is not receptive to the modest fluid loss effects^60^. The amount of fluid ingested, and the time when the fluid was administered has varied physiological responses. These may be confounded in response to dehydration protocol (control and intervention trials) which could have implications on cognitive and athletic performance.

## Conclusion

Considering that, most of the studies measured up to 2.7% body mass loss, the impact of hypohydration and fluid balance on cognitive performance in individual and team sports remains equivocal. In all the studies involved, measures of cognitive function altered include processing speed, vigilance, and reaction time for working memory. It is important to note that visuomotor reaction, mental concentration, and visual scanning and/ perception were not significantly affected by fluid balance and hypohydration. This inconsistency should inform the need to consider objectivity, subjectivity, validity, reliability, and sensitivity of cognitive function assessment tools for the athletic population. The current review serves to draw attention to areas for future research.
